# Microbial Diversity of a Camembert-Type Cheese Using Freeze-Dried Tibetan Kefir Coculture as Starter Culture by Culture-Dependent and Culture-Independent Methods

**DOI:** 10.1371/journal.pone.0111648

**Published:** 2014-10-31

**Authors:** Jun Mei, Qizhen Guo, Yan Wu, Yunfei Li

**Affiliations:** Department of Food Science and Technology, School of Agriculture and Biology, Shanghai Jiao Tong University, Shanghai, P.R. China; University Hospital of the Albert-Ludwigs-University Freiburg, Germany

## Abstract

The biochemical changes occurring during cheese ripening are directly and indirectly dependent on the microbial associations of starter cultures. Freeze-dried Tibetan kefir coculture was used as a starter culture in the Camembert-type cheese production for the first time. Therefore, it's necessary to elucidate the stability, organization and identification of the dominant microbiota presented in the cheese. Bacteria and yeasts were subjected to culture-dependent on selective media and culture-independent polymerase chain reaction (PCR)-denaturing gradient gel electrophoresis (DGGE) analysis and sequencing of dominant bands to assess the microbial structure and dynamics through ripening. In further studies, kefir grains were observed using scanning electron microscopy (SEM) methods. A total of 147 bacteria and 129 yeasts were obtained from the cheese during ripening. *Lactobacillus paracasei* represents the most commonly identified lactic acid bacteria isolates, with 59 of a total of 147 isolates, followed by *Lactococcus lactis* (29 isolates). Meanwhile, *Kazachstania servazzii* (51 isolates) represented the mainly identified yeast isolate, followed by *Saccharomyces cerevisiae* (40 isolates). However, some lactic acid bacteria detected by sequence analysis of DGGE bands were not recovered by plating. The yeast *S. cerevisiae* and *K. servazzii* are described for the first time with kefir starter culture. SEM showed that the microbiota were dominated by a variety of lactobacilli (long and curved) cells growing in close association with a few yeasts in the inner portion of the grain and the short lactobacilli were observed along with yeast cells on the exterior portion. Results indicated that conventional culture method and PCR-DGGE should be combined to describe in maximal detail the microbiological composition in the cheese during ripening. The data could help in the selection of appropriate commercial starters for Camembert-type cheese.

## Introduction

The main focus of research on cheese production in the past two decades has been on the improvement of quality characteristics and the production of healthier cheese [1]. An appropriate starter culture becomes increasingly important in cheese manufacturing and affects biochemical changes occurring during cheese ripening. The biochemical changes, including the metabolism of proteolysis, lipolysis and glycolysis, lead to the formation of key flavor and aroma components of cheese [2], [3]. Consequently, an upsurge of interest in developing suitable starter cultures in cheese production has occurred. Many researchers have proposed a variety of cultures suitable for use as starters, including bifidobacteria, lactococcus, lactobacillus, leuconostoc and enterococcus species [4]–[9].

Recently, the kefir culture has gained researchers' attention with regarding to cheese manufacturing due to its potential effect on quality, health, and safety properties of the product. Resembling small cauliflower florets in kefir grains' appearance, they vary in size from approximately 3–30 mm, and contain a complex mixture of acetic acid bacteria, yeasts and lactic acid bacteria that are considered to have probiotic properties [10]–[13]. Lactic acid bacteria that exist in kefir grains have attracted a lot of attention because of their ability to inhibit the development of spoilage and the growth of pathogenic microorganisms, either by the production of lactic acid or by the expression of antimicrobial agents [14], [15]. Kefir has been used as a starter in white pickled cheese [16], hard-type cheese [1], Feta-type cheese [17], and others. However, for the commercial production of cheese, direct use of kefir grains is impractical regarding transportation, storage, and cell dosage. Freeze-drying is a solution for long-term preservation of microorganisms and convenience for shipping [18].

Molecular culture-independent approaches have proven to be powerful tools in providing a more complete inventory of the microbial diversity in cheese [19]. As far as the development of molecular technology, PCR-DGGE has recently been shown to be a useful tool for studying community structure at the species level. 16S rDNA fragments from different microbial species have the same length but different DNA sequences therefore the species can be identified by the band positions on the DGGE gel. DGGE allows the simultaneous analysis of multiple samples and the comparison of microbial communities based on temporal and geographical differences [20]. Now PCR-DGGE has successfully been applied to analyze the microflora in various foods, such as chilled pork, wine, raw milk, dry fermented sausages, and so on [21]–[24].

The freeze-dried Tibetan kefir coculture has not been tested yet in Camembert-type cheese production where ripening periods are necessary for the product to acquire its microbial diversity. Therefore, the motivation of the present work was to elucidate the stability, organization and identification of the dominant microbiota present in the cheese using freeze-dried Tibetan kefir coculture as starter culture by culture-dependent and culture-independent methods.

## Materials and Methods

### Production of freeze-dried Tibetan kefir coculture

Tibetan kefir coculture isolated from a commercial Tibetan kefir beverage was used in the present study. It was grown on a synthetic medium [17] consisting of 4% lactose, 0.4% yeast extract, 0.1% (NH_4_)_2_SO_4_, 0.1% KH_2_PO_4_, and 0.5% MgSO_4_·7H_2_O at 30°C. The synthetic medium was sterilized at 121°C for 20 min prior to use. Pressed wet-weight cells (about 0.5 to 1.0 g dry weight) were prepared and used directly in aerobic fermentations of whey for further production of kefir coculture. A kefir coculture was resuspended in the fermented whey and the whole suspension was freeze-dried overnight in a freeze-drying system (Free Zone Triad Cascade Benchtop Freeze Dry System, Labconco, Kansas City, U.S.A.).

### Cheese Making

Pilot-scale cheese production (coagulation, cutting, draining, and molding of the curd) was carried out under aseptic conditions in a sterilized, 2 m^3^ (2 m×1 m×1 m) cheese-making chamber in Technical Centre of Bright dairy & Food Co. The cheeses were made according to Leclercq-Perlat et al [25]. Then the cheeses were transferred to the ripening chamber and kept at 14±1°C with 85±2% RH, which was designated as the initial ripening time (day 0). After 24 h, the cheeses were changed to keep at 12±1°C and 95–97% RH for 14 days and at 4°C until day 35. Samples of mature cheese (0, 5, 10, 15, 25 and 35 days old; the date day 35 at which cheese is allowed to be sold according to PDO Council specifications) were taken following standard FIL-IDF procedures. The rind (1 mm thick all over the cheese surface) and body of each cheese (inner layer) were separated by the method of Le Graët and Brûlé [26].

### DGGE analysis of the cheese

To sample the cheeses, 5 g cubes from the inside and 5 g strips from the outside were taken. These samples were then homogenized with 40 mL of a 2% (w/v) sterilized sodium citrate solution at 45°C for 1 min. DNA extraction was accomplished by using a commercial kit (SK8233 soil gDNA Miniprep kit; Songon, China) according to the manufacturer's instructions.

The bacterial community DNA was amplified with primers 338fgc (5′-CGC CCG CCG CGC GCG GCG GGC GGG GCG GGG GCA CGG GGG GAC TCC TAC GGG AGG CAG CAG-3′) (the GC clamp is underlined) and 518r (5′-ATT ACC GCG GCT GCT GG-3′) spanning the V3 region of the 16S rDNA gene [27]. The D1 domain of the 26S rRNA gene of yeasts was amplified using the primers NL1-GC (5′-GCG GGC CGC GCG ACC GCC GGG ACG CGC GAG CCG GCG GCG GGC CAT ATC AAT AAG CGG AGG AAA AG-3′) (the GC clamp is underlined) and a reverse primer LS2 (5′-ATT CCC AAA CAA CTC GAC TC-3′), as reported by Cocolin et al. [23]. All GC primers contained a 39 bp GC-clamp sequence at their 5′ end to prevent the complete denaturation of amplicons. PCR was performed in 50 µL reaction volumes using a Taq-DNA polymerase master mix (Songon, China) with 100 ng of each DNA sample as a template and 0.2 mM of each primer. PCR conditions were as follows: 94°C for 4 min, 30 cycles of 94°C for 30 s, annealing at 56°C for 1 min and extension at 72°C for 30 s, followed by a melting curve.

The PCR products were analyzed by DGGE using a Bio-Rad DCode Universal Mutation Detection System (Bio-Rad, Richmond, CA, U.S.A.). Samples were applied to 8% (w/v) polyacrylamide gels in 1×TAE. Optimal separation was achieved with a 30–60% urea-formamide denaturing gradient (100% correspondent to 7 M urea and 40% (v/v) formamide). The gels were electrophoresised for 16 h at 60 V. Bands were visualized under UV light after staining with ethidium bromide (0.5 µg·mL^−1^) and photographed.

DNA recovered from each DGGE band was reamplified with the primers 338fgc (5′-ACT CCT ACG GGA GGC AGC AG-3′) and 518r (5′-ATT ACC GCG GCT GCT GG-3′) for bacteria [27] and NL1-GC (5′-GCC ATA TCA ATA AGC GGA GGA AAA G -3′) and LS2 (5′-ATT CCC AAA CAA CTC GAC TC-3′) for yeasts [23]. DGGE bands were excised with a sterile scalpel and eluted in 30 mL sterile water, overnight at 4°C to allow diffusion of the DNA. Two microliters of the DNA of each DGGE band was reamplified as described above. The PCR amplicons were then sequenced (Applied Biosystems, Foster City, CA, USA). Sequences were used as a query sequence to search for similar sequences from GenBank by means of the blast program (http://www.ncbi.nlm.nih.gov/BLAST/) [28]. Sequences showing 97% similarity or higher were deemed to belong to the same species [29].

### Culture-dependent approach

Bacteria and yeasts were enumerated by method of Magalhães et al [30] with some modifications. Representative 10g portions of duplicate samples taken from the cheese interior and outside were blended with 90 mL of sterilized Ringer's Solution (1/4 strength) and subjected to serial dilutions.

The following microbiological analyses were performed: (i) determination of total mesophilic bacteria on nutrient agar medium (Huankai, China) at 28°C for 48 h; (ii) enumeration of lactobacillus after incubation on acidified MRS agar (Huankai, China) at 37°C for 48 h anaerobically (Xinmiao YQX-II anaerobic incubator, China); (iii) enumeration of lactococcus after incubation on M-17 agar (Huankai, China) at 37°C for 48 h; (iv) enumeration of acetobacter after incubation on acetobacter medium (Huankai, China) at 30°C for 48 h; (v) enumeration of yeasts after incubation on PDA agar (Huankai, China) containing 100 mg chloramphenicol and 50 mg chlortetracycline (pH adjusted to 4.5 by sterile solution of 10% lactic acid) at 28°C for 5 days. All media for bacterial enumeration were supplemented with 0.4 mg/mL nystatin (Sigma-Aldrich, USA). Gram staining and catalase tests were performed for confirmation of lactic acid bacteria. Results are presented as the log of the mean number of CFU on solid-medium culture plates containing between 30 and 300 colonies per g of cheese.

### Observation of Tibetan kefir grains using SEM

Tibetan kefir grains were sliced to produce samples for microscopy according to Seydim et al. [31]. Samples were collected from the exterior and inner part. The grains were fixed (2.5% glutaraldehyde solution) at 4°C for 24 h. The grains were then transferred to 30% glycerol for 30 min and immersed in liquid nitrogen for subsequent fracture in the metal surface. The grains were post-fixed in 10 g/L osmium tetroxide in phosphate buffer for 1 h at 25°C and dehydrated in alcohol: 15%, 30%, 50%, 70%, 90% and 100%. Prior to observation, the grains were coated with a thin conducting layer of gold. Microstructure observations of the samples were carried out using a FEI Sirion 200 environmental scanning microscope (FEI Company, the Netherlands).

## Results

### Enumeration and identification of isolates by a culture-dependent method

In order to establish the different species of bacteria and yeasts present during ripening, a representative number of isolates from each culture medium were identified (Table 1). Lactococcus and lactobacillus were the most frequently found microorganism and showed an increase from 6.34 to 8.28 logCFU/g and 6.11 to 8.04 logCFU/g until day 35, respectively. The average number of yeasts, which was about 5.34 logCFU/g at day 0, increased to 7.17 logCFU/g at the end. Acetobacter and total mesophilic aerobic bacteria showed similar behavior for growth, ranging from about 5.00 to 7.00 logCFU/g. In general, lactic acid bacteria were much more numerous (10^8^–10^9^) than yeasts (10^5^–10^7^) and acetic acid bacteria (10^5^–10^6^) in the cheese during ripening.

**Table 1:** Mean counts (logCFU/g) of microorganisms in the Camembert-type cheese during ripening using freeze-dried Tibetan kefir coculture as starter.

Time (d)	TMAB	LB	LC	AC	YM^5^
0	5.09+0.07^a^	6.11+0.07	6.34+0.08	5.03+0.07	5.34+0.16
5	5.81+0.11	6.69+0.07	7.05+0.05	5.52+0.14	5.89+0.09
10	6.10+0.06	7.08+0.07	7.57+0.11	6.01+0.06	6.37+0.11
15	6.44+0.10	7.76+0.09	7.94+0.07	6.47+0.09	6.86+0.08
25	6.68+0.09	7.94+0.05	8.10+0.04	6.62+0.16	6.96+0.05
35	6.85+0.06	8.04+0.08	8.28+0.05	6.79+0.08	7.17+0.06

a Values reported are the means ± standard deviations.

5 TMAB, total mesophilic aerobic bacteria; LB, Lactobacillus; LC, Lactococcus; AC, Acetobacter; YM, Yeasts.

A total of 276 isolates were obtained from the cheese (Table 2). Among the isolates, 147 isolates were bacteria and 129 isolates were yeasts. The bacteria contained lactic acid bacteria (104 isolates) and acetic acid bacteria (43 isolates). The culture-dependent approach indicated that *Lactobacillus paracasei* represents the largest and most commonly identified lactic acid bacteria isolates, with 49 of a total of 104 isolates, followed by *Lactococcus lactis* (31 isolates) and *Lactobacillus kefiri* (19 isolates). Isolates of *Lactobacillus kefiranofaciens* (5 isolates) was also sporadically identified. The only acetic acid specie, *Acetobacter lovaniensis*, was also identified (43 isolates). The lactose-fermenting yeasts (*Kluyveromyces lactis*) together with non-lactose-fermenting yeasts (*Trichosporon moniliiforme*, *Debaryomyces hansenii*, *Saccharomyces cerevisiae* and *Kazachstania servazzii*) were found in the cheese during the ripening time. The yeast flora of the cheese was dominated by lactose-negative strains. Among them, *K. servazzii* predominated, with 51 of a total of 129 isolates, followed by *S. cerevisiae* (40 isolates). *K. lactis* (20 isolates) and *D. hansenii* (15 isolates) were the other identified members of yeasts. Isolates of *T. moniliiforme* (7 isolates) was also sporadically identified.

**Table 2:** Distribution of bacteria and yeasts isolated during ripening of the cheese by the culture-dependent methods.

Closest relative	Identity (%)	Accession	Ripening time					
			Day 0	Day 5	Day 10	Day 15	Day 25	Day 35
Bacteria								
*Lactobacillus kefiri*	99%	AB362680.1	+(2)	+(*)	+(7)	+(2)	+(4)	+(4)
*Lactobacillus kefiranofaciens*	100%	NC015602.1	+(*)	+(3)	+(*)	+(*)	+(*)	+(2)
*Lactococcus lactis*	100%	NC002662.1	+(4)	+ (6)	+(5)	+(6)	+(5)	+(5)
*Lactobacillus paracasei*	99%	AB368902.1	+(7)	+(12)	+(10)	+(9)	+(6)	+(5)
*Acetobacter lovaniensis*	98%	AB308060.1	+(5)	+(5)	+(7)	+(11)	+(7)	+(8)
Yeasts								
*Trichosporon moniliiforme*	99%	JN805525.1	−(*)	−(*)	'−(*)	+(4)	+(3)	+(*)
*Debaryomyces hansenii*	99%	HF934036.1	−(*)	+(*)	+(4)	+(5)	+(3)	+(3)
*Kluyveromyces lactis*	99%	AJ229069.1	−(*)	+(4)	−(*)	+(10)	+(6)	+(*)
*Saccharomyces cerevisiae*	99%	EU649673.1	+(16)	+(10)	+(9)	+(5)	+(*)	+(*)
*Kazachstania servazzii*	99%	JQ808010.1	+(13)	+(16)	+(11)	+(11)	+(*)	+(*)

−, not detected by PCR-DGGE; +, detected by PCR-DGGE and sequencing of the DNA fragment upon excision from the gel;

* species not isolated by culturing methods; values in parentheses are numbers of colonies detected by culturing.

### DGGE fingerprinting of bacterial and yeast communities

**Figure 1:**
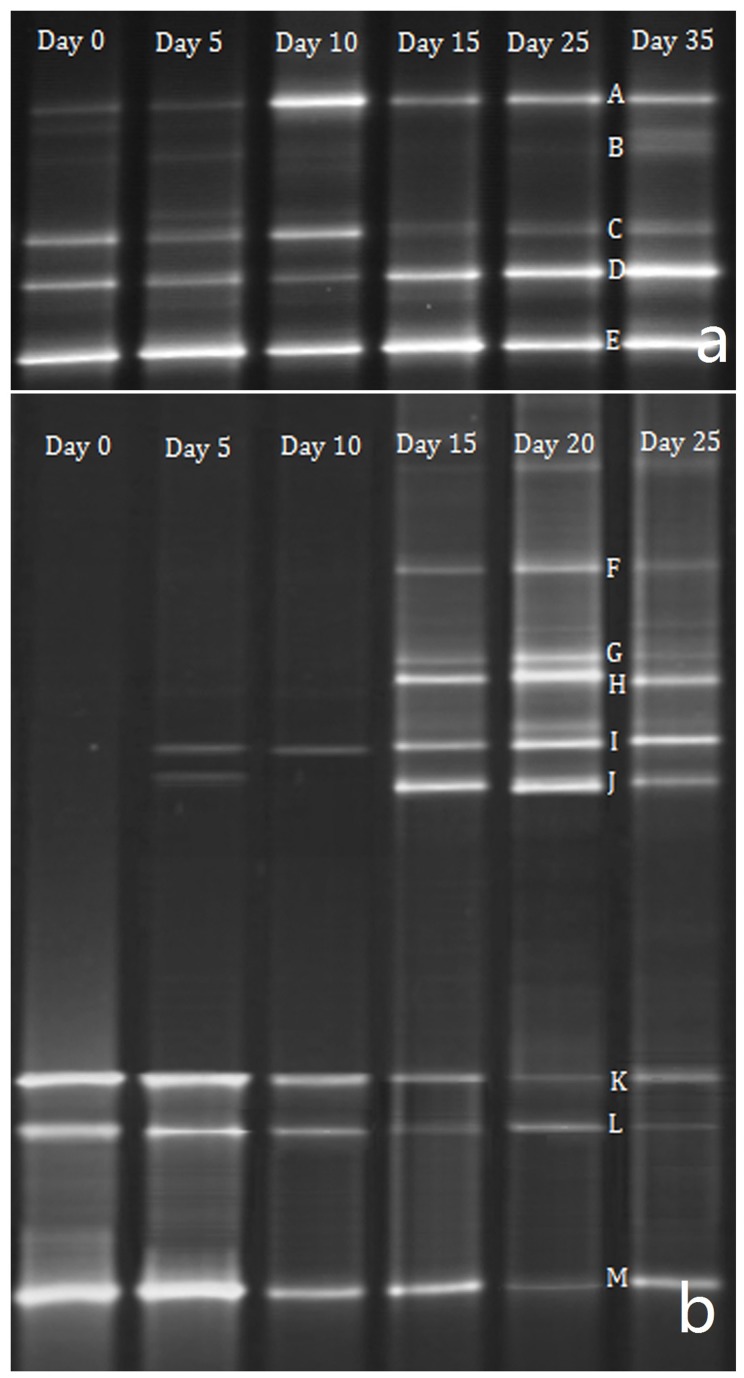
DGGE profiles of bacterial 16S rDNA gene V3 fragments (a) and fungal 26S rRNA gene D1 region (b) amplified from the cheese during ripening.

Traditionally, many culture methods are only partially selective and exclude members of the microbial community. Thus, to determinate the total composition of microbiota in the cheese, PCR-DGGE analysis was used. The V3 region of the 16S rDNA gene of the bacteria and D1 region of the 26S rRNA gene of yeasts were amplified, and representative DGGE fingerprints are shown in Figure 1a and 1b. No differences in community structure during fermentation were found for both bacteria and yeasts. To determine the composition of microbiota, individual bands observed in the DGGE profiles were excised from the acrylamide gel and re-amplified to provide a template for sequencing. After Blast analysis, sequence results showed 98–100% identity with sequences retrieved from GenBank accession numbers. DGGE band A was clearly identified as *Lb. kefiri*, band B as *Lb. kefiranofaciens*, band C as *A. lovaniensis*, band D as *Lc. lactis*, band E as *Lb. paracasei*, band F as *T. moniliiforme*, band H as *Geotrichum candidum*, band I as *D. hansenii*, band J as *K. lactis*, band K as *S. cerevisiae*, band L as *K. servazzii* and band M as *Penicillium crustosum*. Band G was excised from the gel, but it could not be recovered for sequencing. PCR-DGGE analysis showed that species of the genus lactobacillus were the dominant bacteria in the cheese, as already indicated by plating results. The representatives of lactobacillus could be differentiated according to the migration distances of their respective 16S rDNA fragments. *Lc. lactis* was another dominant microbe in the cheese. Band C in the DGGE analysis corresponded to *A. lovaniensis*. Interestingly, this was the only species of non-lactic acid bacteria found by culture-independent methods.

In fungal analysis, PCR-DGGE showed a good correlation with the culture-dependent methods. Band K represented the *Saccharomyces* sensu stricto group (Figure 1b). Among them, *S. cerevisiae* was the most probable strain identified because according to culture-based isolations; this species was the most commonly recovered yeast in the cheese (Table 2). The yeast *S. cerevisiae* and *K. servazzii* are described for the first time in the cheese with kefir starter.

### Distribution of the microflora in the kefir grains as observed using SEM

**Figure 2:**
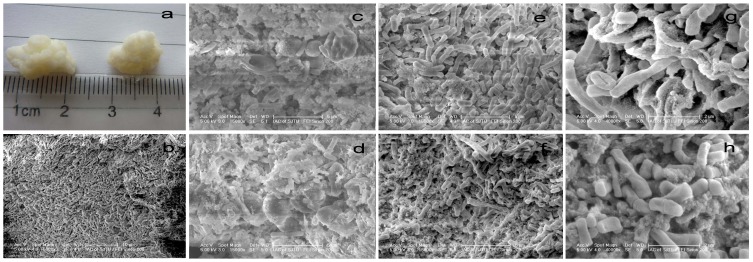
Electron micrographs of Tibetan kefir grains. (a) Tibetan kefir grains; (b) grain surfaces at ×5 000; (c, e) Inner portion of kefir grain at ×15 000; (g) Inner portion of kefir grain at ×40 000; (d, f) Exterior portion of kefir grain at ×15 000; (h) Exterior portion of kefir grain at ×40 000.

The exterior surfaces of the Tibetan kefir grains looked smooth and shiny with the naked eye (Figure 2a). However, the grain surfaces, under SEM at a magnification of ×5000, were revealed to be very rugged (Figure 2b). In the inner portion of the grain (Figures 2c, 2e, ×15 000; 2g, ×40 000), a variety of lactobacilli (long and curved), yeasts and fibrillar material were observed. Kefir grains had a spongy fibrillar structure that was branched and interconnected. Fibrillar material increased progressively towards the interior portions of the grain. On the inner portions of the grain, there were a variety of lactobacilli (long and curved) with only a few yeasts embedded in the fibrillar material. Lactobacilli, yeasts and fibrillar material were also observed at × 15 000 (Figures 2d, 2f) and × 40 000 (Figures 2h) on the exterior portion of the grain. The fibrillar material was most probably the polysaccharide kefiran. The short lactobacilli were observed embedded in the grain along with yeast cells.

## Discussion

Descriptions of the different types of yeasts and bacteria present in cheese or beverage using kefir grains as a starter culture have been provided by different authors [17], [32], [33]. Using conventional culture techniques and culture-independent methods, we have monitored the development of bacterial and yeasts communities in the Camembert-type cheese using freeze-dried Tibetan kefir coculture as starter for the first time.

All the bacteria isolated in this study were Gram-positive and non-motile, except *Acetobacter* which is a Gram-negative bacterium. The groups of lactic acid bacteria cause rapid acidification of the milk through the production of organic acids, mainly lactic acid. Also, their production of acetic acid, ethanol, aroma compounds, bacteriocins, exopolysaccharides, and several enzymes are of importance. In this way, they enhance shelf life and microbial safety, improve texture, and contribute to the pleasant sensory profile of cheese [34]. *Lb. kefiri* is an important bacterium found in the cheese and easily observed from the kefir grains (Figure 2). It can produce NH_3_ from arginine, which could explain the pH value increase in the cheese during ripening. *Lb. kefiranofaciens*, a heterofermentative bacterium, has also been reported as a kefiran (exopolysaccharide) producer [35] and is used as the starter for kefir beverage [36] and Caucasian cultured milk [37]. According to Cheirsilp et al. [37], *S. cerevisiae* could assimilate kefiran production rates of *Lb. kefiranofaciens* in a mixed culture of *Lb. kefiranofaciens* and *S. cerevisiae*. *S. cerevisiae* was also isolated from the cheese. The kefiran has been reported to have antibacterial and antitumor activities, modulate the gut immune system and protect epithelial cells against *Bacillus cereus* exocellular factors and infection [38]. So, the kefiran produced in the cheese could be regarded as one of the mechanisms contributing to health benefits for the consumer. According to Kesmen and Kacmaz, *Lb. kefiranofaciens* was the most dominant species in Turkey kefir grains, while *Lc. lactis* was found to be significantly prevalent in kefir beverages [39]. *Lc. lactis* was also the dominating species in the Camembert-type cheese analyzed in our study. This species was exceeded only by *Lb. paracasei* (Table 2). *Lc. lactis* is of great economic importance because of the world-wide use in cheese making. It is assumed that the lysis of *Lactococcus* during cheese ripening results in the release of intracellular proteolytic and esterolytic enzymes, which contribute to flavor development [40]. *Lb. paracasei* seems to find favorable conditions during the cheese-making process and its presence has already been reported in soft, and in many semi-hard and hard cheeses made from cow, ewe, and goat's milk, such as Caciocavallo [41], Ibores [42], Cheddar [43], and Salers [44]. The fact that *Lb. paracasei* is predominant in cheese is probably linked to its mesophilic properties and antimicrobial properties [45]. It is able to metabolize citrate; this could favor its development [46]. As citrate is gradually consumed in metabolism, *Lb. paracasei* is less detected at the end of ripening. The acetic acid species, *A. lovaniensis*, was also identified in our study. *A. lovaniensis* species belongs to the *A. pasteurianus* group. The species *A. pasteurianus* consists of five subspecies, and *A. pasteurianus* subsp. *lovaniensis* has been also described in fermented food from Indonesian sources [47]. Part of the ethanol produced by yeasts may be converted to acetic acid by the genus *Acetobacter*, which has alcohol dehydrogenase activity and converts ethanol to acetaldehyde [48]. Ethyl esters formed from the esterification of acetic acid and alcohols play quite an important role in cheese flavor.

The main contribution of yeasts to the cheese maturation process is the utilization of lactic acid which in turn increases the pH and therefore favors bacterial growth and initiates the second stage of cheese ripening [49]. Meanwhile, the yeasts partake in microbial interactions and contribute to the formation of aroma precursors such as amino acids and fatty acids [50]. *S. cerevisiae*, which exhibits strong fermentative metabolism and tolerance to ethanol, is known to be superior to non-*Saccharomyces* yeast in the process of alcohol fermentation, as regards Camembert and Blue-veined cheeses [51]. The presence of *S. cerevisiae* in the cheese contributes to enhancement of the organoleptic quality of the cheese, promoting a strong and typically yeasty aroma as well as its refreshing, pungent taste. Acetate esters such as ethyl acetate, isoamyl acetate and isobutyl acetate are mainly synthesized by acetyl-coenzyme A (acetyl-CoA) in *S. cerevisiae*. Since acetyl-CoA is an intermediate in lipid biosynthesis, ester production is closely linked to the metabolism of lipids. Lipids are accumulated at the beginning during cheese ripening and enhance significantly upon the increase of intracellular concentrations of acetyl-CoA [52]. As a result, the acetate esters production is low at the beginning even though *S. cerevisiae* was in a high level. This yeast also reduces the concentration of lactic acid, removes hydrogen peroxide by catalase activity and produces stimulators that stimulate the growth of other bacteria in the cheese [53]. It is also worth noting that the yeast species, *K. servazzii*, detected in this cheese for the first time (from day 0–15), could be associated with the presence of glucose and with the assimilation of some acids produced by lactic acid bacteria. In the present study, *D. hansenii* was frequently isolated from the cheese throughout the ripening period because of its tolerance of high concentrations of NaCl. This finding is in agreement with results obtained for surface-ripened Danish cheese [53]. *D. hansenii* increases pH, which enables the growth of less acid-tolerant coryneform bacteria. *K. lactis* growing on the cheese surface did not ferment lactose and did not therefore produce any lactic acid. At the beginning of ripening the growth of *K. lactis* was considerable. Then the occurrence of proteolysis was correlated with the growth of *K. lactis*, *G. candidum* and *Pen. Crustosum*, added to Camembert cheese production methods, known for their proteolytic and peptidolytic activities. In our study, the growths of *G. candidum* and *Pen. crustosum* was highly related to nitrogen formation. *K. lactis* in the cheese also produces esters through alcoholysis of acyl-CoA and esterification of an organic acid with an alcohol, which impart fruity flavors in cheese [54]. Ethyl acetate is by far the major ester produced by *K. lactis* and also detected in more limited proportions in *G. candidum* [55]. The yeasts isolated were frequently detected during the first 15 days ripening and later decreased, even not detected. Two hypotheses can be postulated, i) competition with the ripening microorganisms, particularly *G. candidum*; ii) inhibition by the fatty acids liberated by *G. candidum* through lipolysis [25]. *T. moniliiforme* assimilates L-methionine as a sole nitrogen source and plays an important role in producing the volatile organic compounds [56].

Based on the results of the fingerprints of the bacterial community and the 16S rDNA sequencing, *L. lactis* was dominant microbe, while no lactococcus was found on SEM, which may be due to the bad attachment of lactococcus. This coincides with the results that Seydim et al. obtained on Turkey kefir using scanning electron microscope [31]. According to our observation, yeasts became less predominant further inside the grain. As reported, other researchers have observed that yeasts predominated at the centre part of granule while the exterior of the grain had mostly lactobacilli and only a few yeasts [57], [58]. They reported a variety of lactobacilli (short, long, and curved) in all parts of the grain samples. However, in our study, long and curved lactobacilli were observed mainly in the interior portions, and short lactobacilli were found mainly in the exterior portions of the grains.

## Conclusions

Based on culture-based detection methods, *Lactobacillus*, *Lactococcus*, *Acetobacter*, and yeasts represented the putative candidates in the cheese. In the latter stage of ripening, some yeasts were less frequently detected due to competition with *G. candidum* and *Pen. crustosum*. The culture based findings were confirmed using DGGE, the dominant microbiotas were composed by yeasts affiliated to *K. servazzii*, *S. cerevisiae*, and bacteria affiliated to *Lb. paracasei, Lc. lactis and A. lovaniensis*. As expected, the combination of culture dependant assays and PCR-DGGE analyses has allowed the microbial ecology of the Camembert-type cheese to be profiled. This application could provide an opportunity to better understand and control the transformation process during cheese ripening. Moreover the profiling of microbial populations occurring in cheese ripening can be useful to determine the technologically important strains to be employed as a suitable starter culture to obtain high quality and safety properties in the final product.
